# Comparative evaluation of mechanical injury methods for establishing stable tracheal stenosis animal models

**DOI:** 10.1038/s41598-024-52230-0

**Published:** 2024-01-29

**Authors:** Hongbin Lin, Mailudan Ainiwaer, Zheng Jiang, Zhenyan Wang, Jun Liu, Fei Chen

**Affiliations:** 1 Department of Otolaryngology-Head and Neck Surgery, West China Hospital, Sichuan University, Chengdu, 610041 Sichuan Province China; 2 Head and Neck Surgical CenterWest China Hospital, Sichuan University, Chengdu, 610041 Sichuan Province China; 3 Department of Otolaryngology-Head and Neck Surgery, The Third People’s Hospital of Sichuan Province, Chengdu, China

**Keywords:** Diseases, Medical research, Pathogenesis

## Abstract

The study aimed to assess the stability of various mechanical injury techniques in creating tracheal stenosis animal models using endoscopic assistance and investigate the viability of tracheal stoma in this process. Twenty-six healthy adult New Zealand white rabbits were randomly assigned to an experimental and control group. The experimental group underwent tracheal incision followed by steel brush scraping with endoscopic assistance, while the control group received nylon brush scraping. Within the control group, two subgroups were formed: Group A underwent scraping without tracheal stoma, and Group B underwent scraping followed by tracheal stoma. Additionally, a sham operation was performed on a separate group without subsequent scratching, resulting in no stenosis formation. Endoscopic observations were conducted at 7, 14, and 21 days post-scraping, followed by histological examinations of euthanized rabbits on the 21st day. Notably, all rabbits in the non-stoma group survived without complications, whereas Group B rabbits faced mortality post-operation. Histological assessments revealed inflammatory cell infiltration, fibroblast proliferation, and collagen fiber deposition in narrowed tracheal specimens. Steel brush scraping with endoscopic assistance proved more effective in inducing stable tracheal stenosis compared to nylon brush scraping. However, the survival challenges of rabbits with tracheal fistula require further investigation.

## Introduction

Tracheal stenosis is a complex condition with diverse causes, leading to restricted airflow and life-threatening breathing difficulties. Factors like endotracheal intubation, prolonged tracheostomy, trauma, airway burns, and laryngotracheal surgery contribute to its development. As advances in respiratory support extend critically ill patients' survival, iatrogenic tracheal stenosis rates have risen^1,2^. Current treatments, such as surgery and medication, face challenges due to surgical trauma, high restenosis rates, and uncertainties in drug efficacy^3,4^. The intricate pathogenesis poses a clinical intervention challenge. Therefore, creating a simple, reliable, and reproducible animal model of tracheal stenosis is a vital research objective, to comprehend its mechanisms and explore effective preventive and therapeutic strategies.

Currently, various methods are available to establish an animal model of tracheal stenosis, such as tracheal intubation^5–7^, CO2 laser^8,9^, semiconductor laser^10^, nylon brush scraping^11^, bleomycin steel brush^12^, and electric drill, among others. Experimental subjects commonly include rats, rabbits, pigs, and dogs. However, each approach has its limitations; large animal use may pose safety concerns, and some methods require substantial costs. Small animals' narrow airways may result in higher mortality rates, making it challenging to reach the experimental endpoint^10,12^. Additionally, even with the same scraping method for modeling, different outcomes have been observed^14,15^. In this study, our aim is to establish an animal model of tracheal stenosis that is cost-effective, low in mortality, and exhibits high stability. To achieve this, we employed an open mechanical injury approach for modeling, comparing different scraping methods' outcomes, and providing a detailed account of crucial steps to attain stable stenosis during the modeling process.

## Materials and methods

### Materials

#### Experimental animals

In this study, 26 healthy adult New Zealand white rabbits, weighing 2.5–3.0 kg, were selected as experimental subjects. The experimental procedures were approved by the Animal Ethics Committee of West China Hospital, Sichuan University, and all operations complied with the regulations for the management of experimental animals. The study is reported in accordance with ARRIVE guideline. The sex of the rabbits was not determined.

#### Reagents and medications

The following reagents and medications were used: 3% Pentobarbital sodium; 0.1% Epinephrine; 2% Lidocaine hydrochloride; Penicillin sodium; 0.9% saline.

#### Instruments and equipment

The following instruments and equipment were employed: 3.9 mm otolaryngology endoscope; 3.5 mm nylon fiber brush; 3.5 mm steel brush (Bristle length: 50 mm, core diameter: 3.5 mm, item catalog number: 00355. Jiaji Brush Corporation, Anhui, China.); Electric suction apparatus; Optical microscope; V70 electrocoagulation device.

### Methods

#### Experimental procedure

Before the experiment, all animals underwent an 8-h fasting period without water access. Thirty minutes before the operation, the penicillin was given intramuscularly at a dose of 20000 U/kg, and anesthesia was induced through intravenous injection of a 3% pentobarbital solution at a dose of 1–1.5 ml/kg via the auricular vein. The rabbits were then fixed supine on the operating table, and the anterior neck area's hair was shaved. The neck area was sterilized with 0.5% povidone-iodine, and sterile drapes were applied. Local anesthesia was administered using a 2% lidocaine hydrochloride solution along the midline of the anterior neck to minimize pain. A 3 cm longitudinal incision was made on the skin, gradually exposing the tracheal rings and trachea while carefully avoiding important blood vessels and nerves in the neck. Hemostasis during the operation was achieved using electrocoagulation. For the experimental group, a transverse incision was made on the trachea at the level of the 10th tracheal ring, cutting approximately 2/3 of the tracheal circumference without damaging the tracheal mucosa. In case of bleeding at the cut end of the trachea, epinephrine-soaked cotton was used for hemostasis, and an electric suction apparatus prevented blood from entering the trachea, to prevent mucosal damage caused by suction devices, the suction force was restricted at a level not exceeding 0.02 MPa. The trachea was gently pulled upward with the left hand, transforming the C-shaped cartilage into an O-shape. Then, a 3.5 mm steel brush was inserted into the proximal end of the trachea, rotating and scraping against the tracheal wall ten times over a length of approximately 1 cm to remove blood clots and secretions. Epinephrine-soaked cotton was used for hemostasis in the trachea. Following scraping, an endoscope was inserted through the oral cavity to observe and record the tracheal wall's condition. Throughout the process, ensuring unobstructed airflow in the proximal end of the trachea was paramount to prevent asphyxiation of the experimental rabbits.

**Figure 1:**
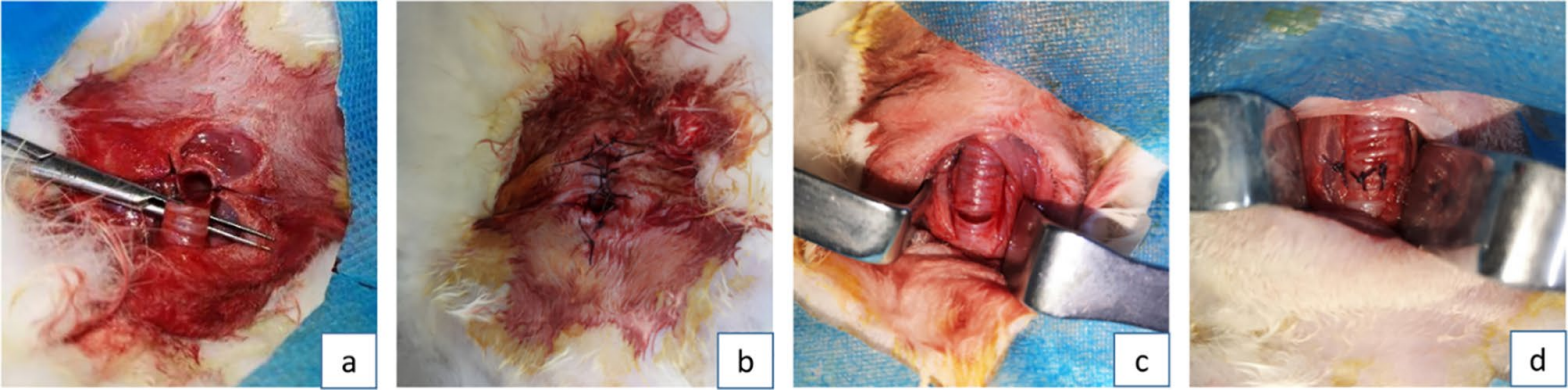
Different ways of managing the trachea after modeling: (**A**) and (**B**) involved complete transection and fistulization of the trachea following modeling. (**C**) and (**D**) entailed intermittent closure of the trachea without fistulization post-modeling.

After complete scraping and hemostasis, the trachea was sutured with 4–0 silk thread using simple interrupted suture technique and repositioned to its original location. The muscle, subcutaneous tissue, and skin were sutured in layers. For control group A, a nylon brush of the same diameter was used for scraping, following the same method as the experimental group. However, no tracheal stoma was created after the surgery. In control group B, the posterior tracheal wall was separated from the esophagus at the 10th tracheal ring, and the trachea was completely transected at that ring. The same scraping method was applied using the nylon brush, and the muscle and subcutaneous tissues were not sutured. Instead, the proximal and distal ends of the trachea were directly sutured to the skin to create a tracheal stoma (see Fig. 1). The sham operation group underwent a tracheotomy without subsequent scraping or irritation; thereafter, immediate closure of the trachea, subcutaneous tissue, and skin was performed.

#### Monitoring of the post-modeling conditions

Postoperatively, daily observations were conducted on the experimental rabbits' mental status, dietary intake, and activity levels, monitoring for signs such as wheezing, breathing difficulties, and any other symptoms.

#### Endoscopic observation and stenosis calculation

All experimental rabbits underwent endoscopy at 7 days, 14 days, and 21 days after the scraping procedure. Tracheal stenosis degree was calculated at the 21-day mark. Each observation occurred under general anesthesia, capturing images via the endoscope. For accurate image acquisition, the endoscope's distal end was positioned at the stenosis start, just above a normal tracheal ring, for photography. Adobe Photoshop was used to analyze and measure the endoscopy images; pixel values was indicated as the measurement index to calculate the maximal diameter (D) and minimal diameter (d) of the trachea before surgery and after stenosis formation.

The calculation of the degree of stenosis followed the method described by Nakagishi^11^. The formula for calculation was (1−s/S) × 100%, where "s" represents the area of the narrowest tracheal portion when stenosis occurred, and "S" represents the area of the lumen enclosed by the cartilage rings. The value of "S" can be calculated as follows: S = π[(D + d)/2]^2^, where "D" and "d" are the maximum and minimum diameters of the lumen enclosed by the cartilage rings, respectively. The same method was used to calculate the value of "s".

#### Gross and histopathological observation

At the 21-day mark, surviving experimental rabbits were euthanized with an overdose of anesthesia. The most narrowed tracheal specimens were excised to observe the type of stenosis and then they were fixed in 10% neutral formalin solution for 24 h. Following routine dehydration with ethanol, tissue blocks were embedded in paraffin, sectioned, and subjected to hematoxylin and eosin staining and Masson's staining. Microscopic analysis focused on changes in the mucosal epithelial layer, fibroblasts, collagen fibers, and inflammatory cells. Rabbits that died during the experiment underwent immediate autopsy to ascertain the cause of death.

#### Statistical analysis

The degree of stenosis obtained during the experiment was statistically analyzed using SPSS 29.0 software. Independent sample t-tests were used, with a significance level set at p < 0.05 indicating statistical significance.

### Ethical consideration

The experimental procedures were approved by the Animal Ethics Committee of West China Hospital, Sichuan University.

## Results

### Modeling results

**Figure 2:**
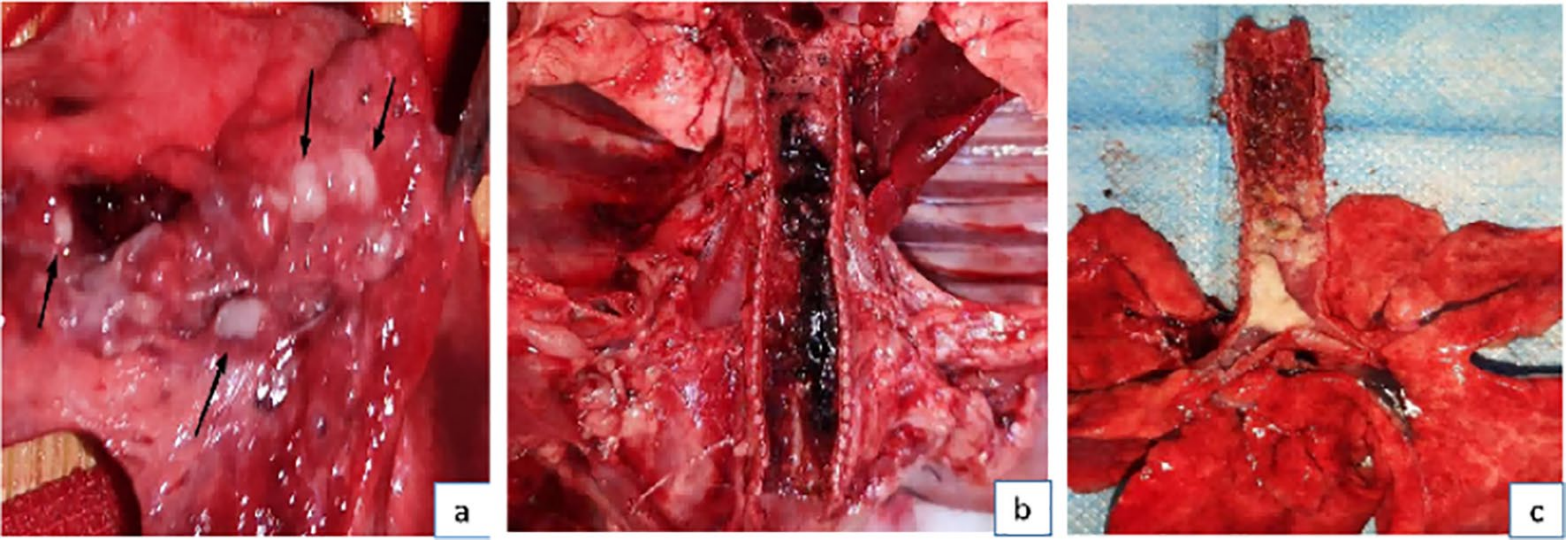
Post-mortem examination of rabbits. (**a**) Lung Infection: The black arrows indicate purulent secretions within the trachea, suggesting lung infection. (**b**) Tracheal Blood Clots and Scabs: Visible blood clots and scabs inside the trachea, indicating the healing process. (**c**) Tracheal Obstruction: The tracheal lumen shows blockage due to accumulated secretions and scabs.

All experimental rabbits without tracheal stoma creation survived throughout the observation period. The nylon-fiber-scraped group and the sham operation group exhibited good mental and activity states throughout the entire experimental period, displaying normal water and food intake without any observed wheezing. However, the steel-brush-scraped group manifested wheezing three days postoperatively, persisting throughout the observation period, accompanied by a decline in mental and activity states. Although food and water intake slightly decreased, by the seventh-day post-surgery, these measures gradually returned to normal alongside improved activity levels. Conversely, those subjected to tracheal stoma creation exhibited varying mortality rates after the surgery, with none surviving until the end of the observation period. Furthermore, these rabbits experienced reduced food intake and deteriorated mental state post-surgery. Immediate autopsy and pathological examination of the deceased rabbits revealed significant findings: (1) One experimental rabbit perished 56 h after surgery due to rupture of blood vessels in the proximal trachea, leading to hemoptysis and asphyxiation. Concurrently, this rabbit developed a lung infection. (2) Four other experimental rabbits also succumbed, with their airways obstructed by scabs and secretions, causing asphyxiation due to blockage in the proximal trachea. However, there was no evidence of lung infection in these cases (see Fig. 2).

### Endoscopic findings and calculation of tracheal stenosis

**Figure 3:**
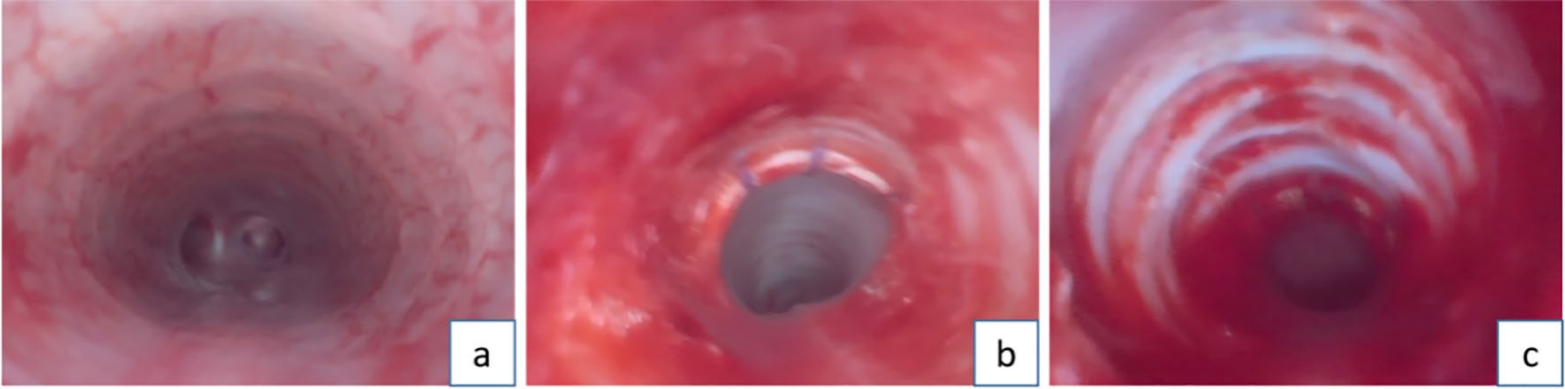
Comparison of Tracheal Laryngoscopic Images Before and After Different Scraping Methods. (**a**) Normal Tracheal Mucosa. (**b**) Tracheal Mucosa After Nylon Brush Scraping: The mucosa exhibits congestion and swelling but remains intact. (**c**) Tracheal Wall After Steel Brush Scraping: Image revealing the complete detachment of the tracheal mucosa after ten scrapings with the steel brush. The tracheal cartilage is clearly visible.

**Figure 4:**
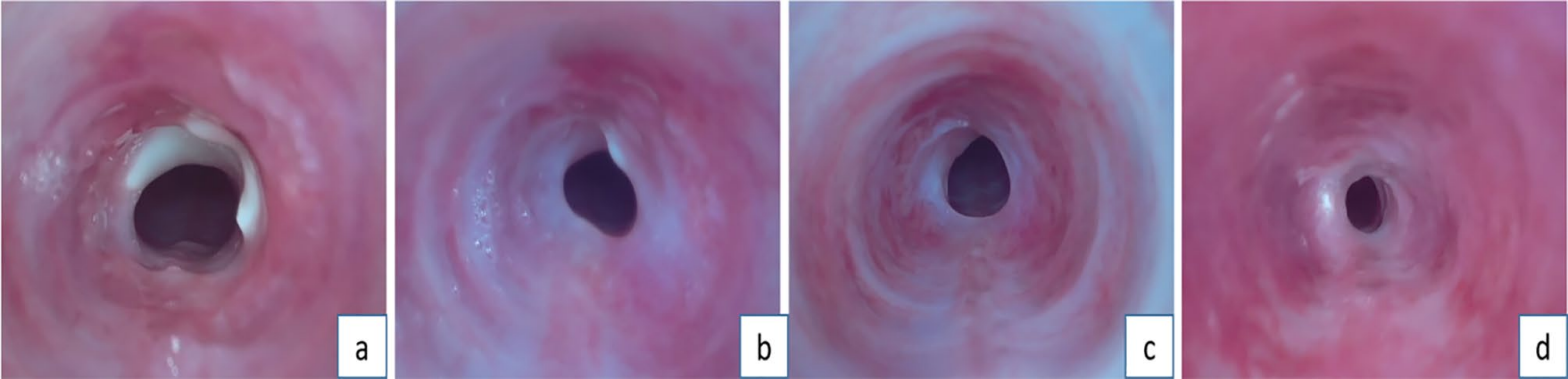
Tracheal Endoscopic Findings at Different Time Points After Steel Brush Scraping. (**a**) Tracheal Endoscopy at 1 Week After Steel Brush Scraping: The tracheal lumen shows early signs of narrowing. (**b**) Tracheal Endoscopy at 2 Weeks After Steel Brush Scraping: Tracheal narrowing is more pronounced compared to the previous week. (**c**,**d**) Tracheal Endoscopy at 3 Weeks After Steel Brush Scraping: The tracheal lumen exhibits a significant degree of stenosis.

Immediate postoperative endoscopy revealed that after ten scrapings with the nylon brush, the tracheal mucosa displayed congestion and swelling, but no mucosal detachment occurred. Conversely, ten scrapings with the steel brush resulted in visible mucosal detachment (see Fig. 3). At seven days post-scraping, the steel brush group showed local mucosal swelling and pseudomembrane formation, with evident tracheal narrowing by day 14. By day 21, all rabbits in the steel brush group exhibited significant narrowing, with degrees ranging from 55 to 84% and an average of 69.9% (see Fig. 4). According to the Myer-Cotton grading system, all rabbits in the steel brush group reached at least Grade II stenosis (100%).

**Figure 5:**
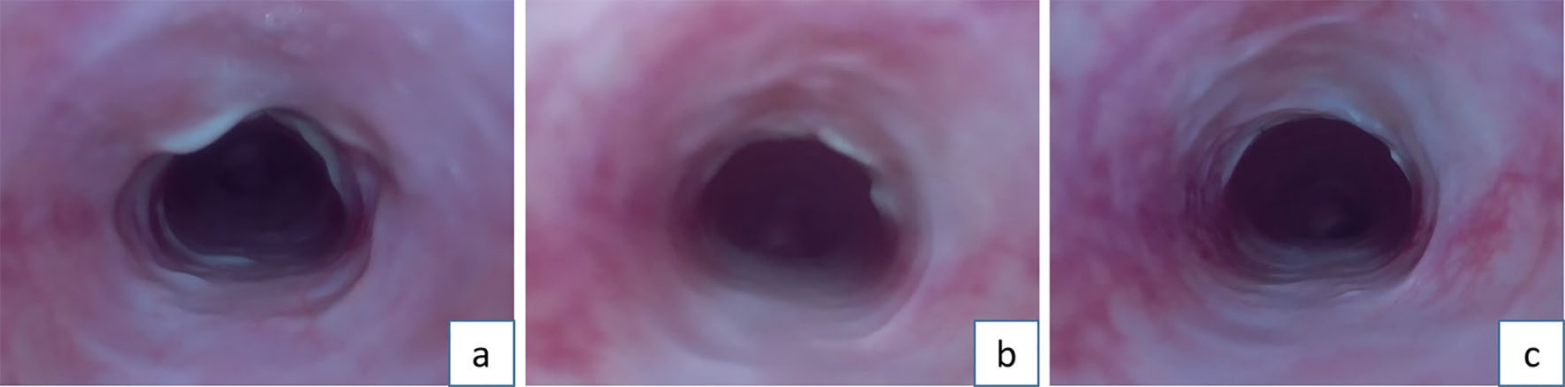
Different timepoint after nylon brush scraping, (**a**) one-week post-surgery; (**b**) two weeks post-surgery; (**c**) three weeks post-surgery, with only mild stenosis formed in the trachea.

**Figure 6:**
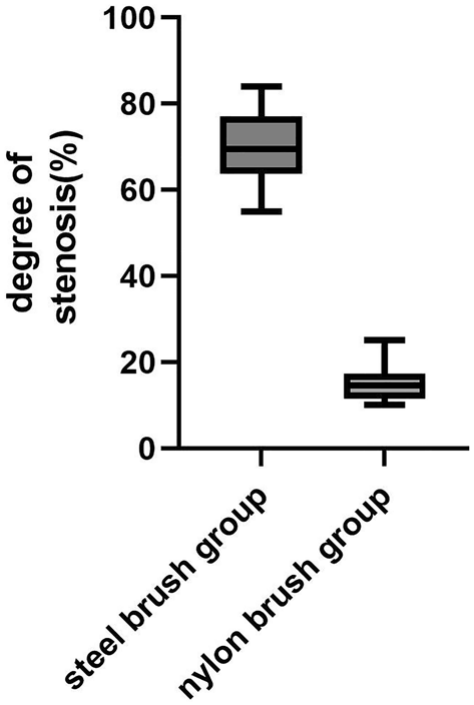
Statistical comparison of tracheal narrowing between the steel brush group and the nylon brush group without tracheal stoma (p < 0.05, n = 8).

Due to the short survival time of rabbits in the tracheal stoma creation group (less than one week), endoscopy was not performed on this group. However, in the group that underwent scraping without tracheal stoma creation using the nylon brush (Group A), only mild narrowing was observed, with degrees ranging from 10 to 25% and an average of 15.1% (see Fig. 5). No stenosis formed in sham operation group. The degree of narrowing in the steel brush group was significantly higher than that in the nylon brush group (Group A), showing a statistically significant difference (p < 0.05) (see Fig. 6).

### Gross and histopathological examination

**Figure 7:**
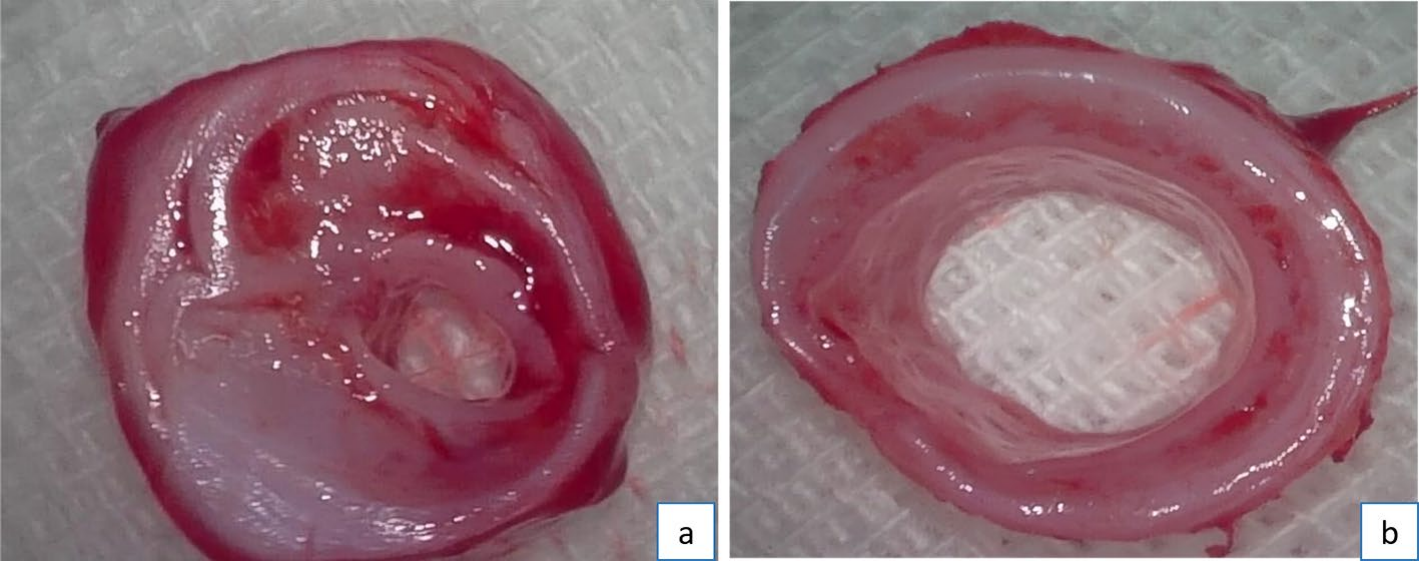
Gross specimen observation: a: remarkable stenosis formation in steel brush scraping group; b: mild stenosis formation in nylon brush group.

**Figure 8:**
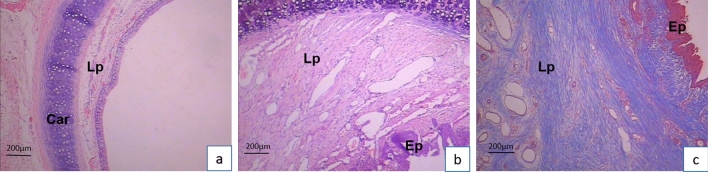
Histopathological Examination Results at 21 Days After the Experiment (Scale bar = 200 μm). (**a**) HE Staining of Trachea After Nylon Brush Scraping. (**b**) HE Staining of Trachea After Steel Brush Scraping. (**c**) Masson's Staining of Trachea After Steel Brush Scraping. Car: Cartilage, Lp: Lamina propria, Ep: Epithelial layer.

We dissected all the subjects that survived the observation period and discovered that the stenosis formation was resulted from granulation tissue hyperplasia. The stenosis was most prominent in steel brush scraping group, which is consistent with endoscopic observation (see Fig. 7). In experimental rabbits surviving until the observation period's end, the narrowest tracheal rings underwent histopathological examination. HE staining indicated notable lamina propria thickening in the steel brush group compared to the nylon brush group. The steel brush group showed a noticeable presence of inflammatory cells, along with fibroblast proliferation and loss of ciliated epithelial cells in the mucosal layer. Masson's staining exhibited abundant collagen fibers within the lamina propria (see Fig. 8).

## Discussion

In recent years, with the gradual advancement of critical care rescue technology, the incidence of iatrogenic tracheal stenosis caused by tracheal intubation has been on the rise^16^. However, the complex pathogenesis of tracheal stenosis has made it a persistent clinical challenge, and current treatment methods have proven difficult to achieve definitive alleviation^17^.

Currently, research on the mechanism of tracheal stenosis is largely limited to animal models. For a long time, the New Zealand White Rabbit has been favored by researchers as the preferred model to study human tracheal stenosis due to its ease of handling, ready availability, safety, and reproducibility. Moreover, the anatomical structure of its larynx and trachea bears remarkable resemblance to that of humans^18^, and the diameter of its trachea is comparable to that of infants and young children^19^. Additionally, some scholars have demonstrated through histopathological examinations of rabbit models at different time intervals after modeling that the characteristics of the injury repair process in rabbits are similar to that in human tracheal stenosis^11,14^. Therefore, we have also chosen this animal as our experimental subject. However, unlike larger animals such as pigs and rabbits, excessive stenosis in rabbits can lead to fatal asphyxia, posing a major challenge in achieving a stable stenosis model while reducing mortality rates.

In this experiment, our objective was to establish a stable tracheal stenosis animal model. This modeling method not only enabled dynamic observation of stenosis changes but also offered a more convenient avenue for administering novel treatments, including topical medications. Regrettably, all rabbits with tracheal stoma succumbed to varying time intervals post-surgery. Postoperative observations revealed their diminished vitality, reduced food intake, and swallowing challenges, possibly attributed to substantial surgical trauma and esophageal involvement.

Upon examining the deceased rabbits, we noted the buildup of obstructive scabs in the proximal trachea, leading to fatal asphyxiation. Additionally, some rabbits exhibited hemoptysis. These deleterious consequences stem from the altered respiratory pathways following tracheostomy, which impairs the nasal cavity's crucial functions of air heating, humidification, and filtration^20^. Consequently, the tracheal mucosa dries out, promoting scab formation and elevating the risk of local and pulmonary infections.

Prior investigations conducted by Dion GR et al. employed rabbits to create tracheal stomas for studying laryngeal thermal injury in animal models^21^. However, the observation time was limited, and postoperative care details were lacking. Subsequently, the researchers switched to pigs as experimental subjects, enabling the study of dynamic changes in laryngeal thermal injury over an extended duration. In these pig models, a heat and moisture exchange cap was applied to the tracheal stoma site to prevent dryness and scab formation. Tracheal tubes were also inserted, and meticulous nursing measures, including timely suctioning and clearing of obstructions, were implemented. Despite these endeavors, some subjects faced airway complications shortly after surgery^22^. Given the restricted size of the rabbit airway, prolonged tracheal stoma creation may not be appropriate. To extend research on tracheal stoma models, larger airway animals as experimental subjects, along with comprehensive tracheostomy care, may be more suitable.

Tracheal stenosis caused by endotracheal intubation primarily results from prolonged intubation, leading to mucosal necrosis, sloughing, and exposure of the cartilage^23^. In some cases, this can even lead to chondritis and chondronecrosis, ultimately causing abnormal healing and subsequent stenosis^24^. Although tracheal stenosis models created by endotracheal intubation best simulate the pathological process seen in clinical tracheal stenosis patients, these models are mostly implemented in large animals such as pigs and dogs^5,6,22^. However, the safety concerns and high costs associated with these experiments limit the wide application of this model.

In 2005, Nakagishi et al. proposed a novel method using New Zealand white rabbits to establish models through tracheal incision followed by scraping with nylon brushes. After 10 scrapings, consistent stenosis with a degree ranging from 29 to 87% was achieved, demonstrating that this approach could effectively mimic the pathological changes seen in human cases^11^. This simple and cost-effective method gained favor among many researchers, although it yielded varied results. Zhang et al. attempted a similar modeling technique but using a 5.5 mm nylon brush for 20 scrapings, resulting in a stenosis rate of only 28.57%^25^. On the other hand, Steehler et al. utilized a 5.0 mm nylon brush for scraping yet failed to induce stable stenosis^15^. Hence, it is evident that nylon brush scraping does not always lead to consistent stenosis. However, when the cartilage was damaged during scraping, the stenosis rate could reach 100%, with a degree ranging from 50.59 to 87.42%^25^. This observation aligns with the viewpoint of most researchers in recent years, suggesting that tracheal stenosis formation in animal models is not directly proportional to the width of the injury but significantly associated with its depth, with more substantial injuries reaching the cartilage layer being more likely to cause stenosis^10,15,25–27^.

The failure to induce stenosis through nylon brush scraping may be attributed to multiple factors. Firstly, the short diameter of the nylon brush may have limited its complete contact with the tracheal mucosa. Secondly, the limited number of scrapings might have hindered stenosis formation^15^. However, the primary reason appears to be the inadequate injury inflicted by the nylon brush, despite multiple contacts with the tracheal wall, as it could not effectively cause mucosal sloughing. In contrast, the steel brush offers significantly improved hardness over nylon brushes. With 2–3 scrapings, the mucosa could be readily removed, allowing subsequent scrapings to injure the cartilage membrane and cartilage. Studies using laser modeling reported mortality rates of up to 60% for circumferential tracheal injuries^10^. However, when only the anterior and lateral walls of the trachea were injured, leaving the posterior wall intact, the survival time could be significantly prolonged^28^. Hence, during the procedure, we elevated the distal trachea with the left hand, gently transforming the tracheal cartilage from a C-shaped configuration to an O-shaped one, causing the membranous tracheal wall to protrude posteriorly. This approach not only prevented damage to the membranous tracheal wall but also ensured sufficient contact between the steel brush and the tracheal cartilage, facilitating the formation of stable stenosis and reducing the mortality rate.

Despite involving open surgery, we restored the rabbits' normal respiratory pathways postoperatively, resembling an in-situ model. The rabbits resumed normal drinking and eating without analgesics, reducing confounding factors from medications. This approach allowed stable stenosis establishment without perioperative deaths, observing all rabbits until the endpoint. The steel brush scraping modeling successfully created stable and reliable stenosis, providing a robust animal model for future tracheal stenosis studies.

However, the study has limitations, including a small sample size and the lack of dynamic observation of pathological changes, hindering comprehensive understanding of the post-injury repair process. Subsequent experiments should aim to improve these aspects. Unfortunately, none of the animal models with tracheal stoma survived, failing to establish a perfect model that is able to largely facilitate monitoring and medication application, further exploration is needed in the future. However, this modeling approach efficiently induces stenosis without resulting in mortality. It can serve as a valuable model for investigating the mechanisms underlying tracheal stenosis and its treatment options in subsequent studies.

## Conclusion

The use of a steel brush, with its superior hardness, enables easy tracheal mucosal injury and damage to the cartilage membrane, leading to stable stenosis without an increase in perioperative mortality. Therefore, the steel brush scraping method is highly recommended for mechanical injury studies.

## Data Availability

The data that support the findings of this study are available from the corresponding author upon reasonable request.
